# Crystal structure of pyriproxyfen

**DOI:** 10.1107/S2056989015013481

**Published:** 2015-07-22

**Authors:** Gihaeng Kang, Jineun Kim, Hyunjin Park, Tae Ho Kim

**Affiliations:** a Department of Chemistry and Research Institute of Natural Sciences, Gyeongsang National University, Jinju 660-701, Republic of Korea

**Keywords:** crystal structure, pyriproxyfen, ether, juvenile hormone mimic, insecticide, π–π stacking

## Abstract

In the title compound {systematic name: 4-phen­oxy­phenyl (*RS*)-2-[(pyridin-2-yl)­oxy]propyl ether}, C_20_H_19_NO_3_, which is a juvenile hormone mimic and insecticide, the dihedral angles between the plane of the central benene ring and those of the pendant pyridine ring and phenyl ring are 78.09 (6) and 82.14 (8)°, respectively. The conformation of the O—C—C—O linkage is *gauche* [torsion angle = −75.0 (2)°]. In the crystal, weak aromatic π–π stacking inter­actions [centroid–centroid separation = 3.8436 (13) Å] and C—H⋯π inter­actions link adjacent mol­ecules, forming a three-dimensional network.

## Related literature   

For information on the insecticidal properties of the title compound, see: Shah *et al.* (2015 ▸). For related crystal structures, see: Ji *et al.* (2013 ▸); Kang *et al.* (2014 ▸).
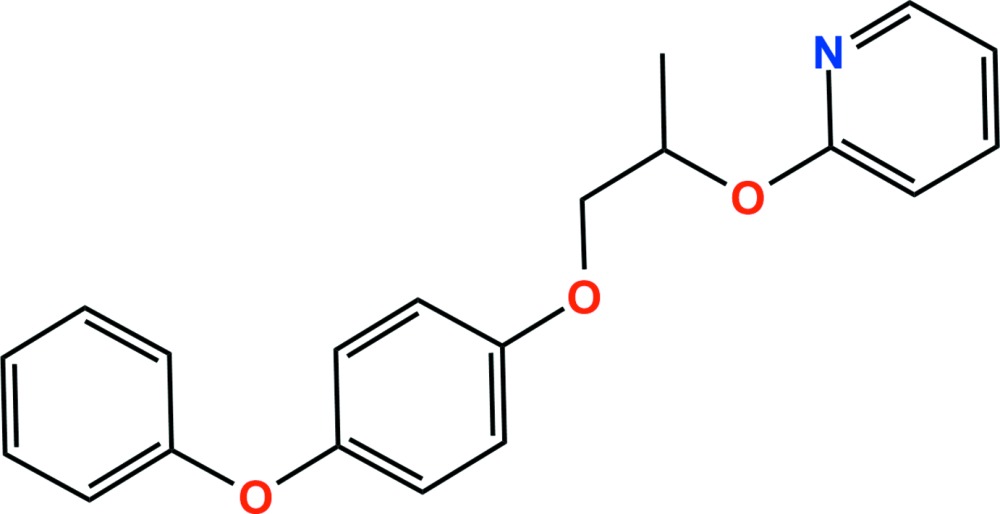


## Experimental   

### Crystal data  

C_20_H_19_NO_3_
*M* *_r_* = 321.36
Orthorhombic, 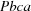

*a* = 10.0676 (2) Å
*b* = 8.0279 (1) Å
*c* = 40.9129 (7) Å
*V* = 3306.65 (10) Å^3^
*Z* = 8
Mo *K*α radiation
μ = 0.09 mm^−1^
*T* = 173 K
0.25 × 0.13 × 0.03 mm

### Data collection  

Bruker APEXII CCD diffractometer
Absorption correction: multi-scan (*SADABS*; Bruker, 2009 ▸) *T*_min_ = 0.979, *T*_max_ = 0.997
52074 measured reflections
3238 independent reflections
2515 reflections with *I* > 2σ(*I*)
*R*_int_ = 0.065

### Refinement  

*R*[*F* ^2^ > 2σ(*F* ^2^)] = 0.056
*wR*(*F* ^2^) = 0.148
*S* = 1.04
3238 reflections
218 parameters
H-atom parameters constrained
Δρ_max_ = 0.66 e Å^−3^
Δρ_min_ = −0.25 e Å^−3^

### 

Data collection: *APEX2* (Bruker, 2009 ▸); cell refinement: *SAINT* (Bruker, 2009 ▸); data reduction: *SAINT*; program(s) used to solve structure: *SHELXS97* (Sheldrick 2008 ▸); program(s) used to refine structure: *SHELXL2013* (Sheldrick, 2015 ▸); molecular graphics: *DIAMOND* (Brandenburg, 2010 ▸); software used to prepare material for publication: *SHELXTL* (Sheldrick, 2008 ▸).

## Supplementary Material

Crystal structure: contains datablock(s) global, I. DOI: 10.1107/S2056989015013481/hb7462sup1.cif

Structure factors: contains datablock(s) I. DOI: 10.1107/S2056989015013481/hb7462Isup2.hkl

Supporting information file. DOI: 10.1107/S2056989015013481/hb7462Isup3.cml

**Fig. 1.**
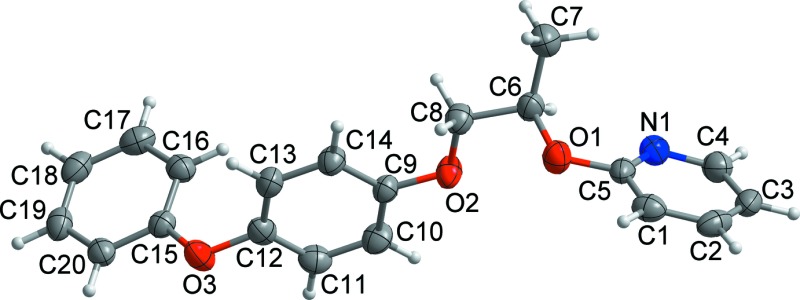
The asymmetric unit of the title compound with displacement ellipsoids drawn at the 50% probability level. H atoms are shown as small spheres of arbitrary radius.

**Fig. 2.**
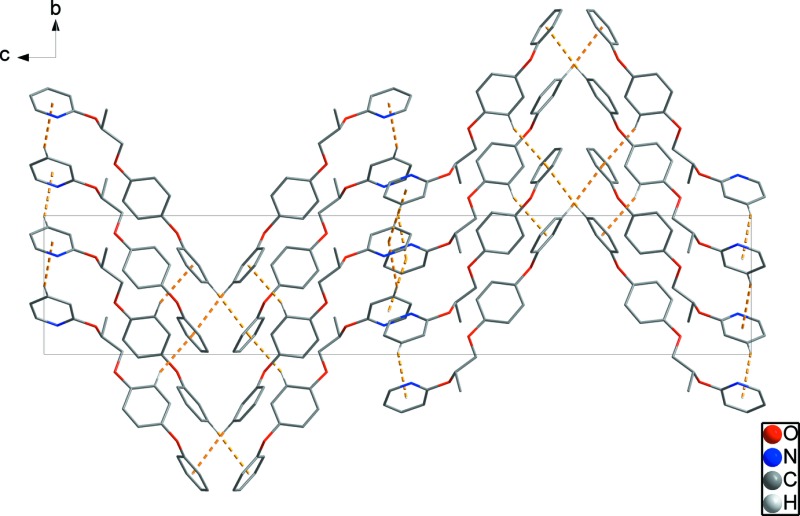
Crystal packing viewed along the a axis. The weak C—H···π and π–π interactions are shown as dashed lines.

. DOI: 10.1107/S2056989015013481/hb7462fig1.tif
The asymmetric unit of the title compound with displacement ellipsoids drawn at the 50% probability level. H atoms are shown as small spheres of arbitrary radius.

a . DOI: 10.1107/S2056989015013481/hb7462fig2.tif
Crystal packing viewed along the *a* axis. The weak C—H⋯π and π–π inter­actions are shown as dashed lines.

CCDC reference: 1412612

Additional supporting information:  crystallographic information; 3D view; checkCIF report

## Figures and Tables

**Table 1:** CH interactions (, ) *Cg*1 and *Cg*2 are the centroids of the N1/C4/C3/C2/C1/C5 and C15C20 rings, respectively.

*D*H*A*	*D*H	H*A*	*D* *A*	*D*H*A*
C2H2*Cg*1^i^	0.95	2.85	3.667(3)	145
C14H14*Cg*2^ii^	0.95	2.86	3.733(2)	152
C19H19*Cg*2^iii^	0.95	2.97	3.857(2)	156

Symmetry codes: (i) 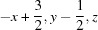; (ii) 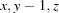; (iii) 
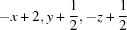
.

## supplementary crystallographic information

### S1. Comment

Pyriproxyfen, [systematic name: 4-phenoxyphenyl (*RS*)-2-(2-pyridyloxy)propyl ether], is the juvenile hormone mimics and it has been used for the management of many insect pests including the house fly (Shah *et al.*, 2015). However, until now its crystal structure has not been reported. In the title compound (Fig. 1), the dihedral angles between the planes of the central benzyl ring and the terminal pyridine ring and phenyl ring system are 78.09 (6) and 82.14 (8)°, respectively. All bond lengths and bond angles are normal and comparable to those observed in the crystal structure of a similar compound (Ji *et al.*, 2013; Kang *et al.*, 2014).

In the crystal (Fig. 2), weak intermolecular C—H···π interactions link adjacent molecules, forming a three-dimensional network (Table. 1). In addition, weak π–π interactions [*Cg*1···*Cg*1^iv^, 3.8436 (13) Å] are present (*Cg*1 is the centroid of the N1–C5 ring)[for symmetry codes: (iv),-*x* + 2,-*y* - 1,-*z*].

### S2. Experimental

The title compound was purchased from the Dr Ehrenstorfer GmbH Company. Slow evaporation of a solution in CH_2_Cl_2_ gave single crystals suitable for X-ray analysis in the form of colourless blocks.

### S3. Refinement

All H-atoms were positioned geometrically and refined using a riding model with d(C—H) = 1.00 Å, *U*_iso_ = 1.2*U*_eq_(C) for C*sp*^3^—H, d(C—H) = 0.99 Å, *U*_iso_ = 1.2*U*_eq_(C) for CH_2_ groups, d(C—H) = 0.98 Å, *U*_iso_ = 1.2*U*_eq_(C) for CH_3_ groups, d(C—H) = 0.95 Å, *U*_iso_ = 1.2*U*_eq_(C) for aromatic C—H.

### Crystal data

**Table:**

C_20_H_19_NO_3_	*D*_x_ = 1.291 Mg m^−^^3^
*M**_r_* = 321.36	Mo *K*α radiation, λ = 0.71073 Å
Orthorhombic, *P**b**c**a*	Cell parameters from 7580 reflections
*a* = 10.0676 (2) Å	θ = 2.3–22.0°
*b* = 8.0279 (1) Å	µ = 0.09 mm^−^^1^
*c* = 40.9129 (7) Å	*T* = 173 K
*V* = 3306.65 (10) Å^3^	Block, colourless
*Z* = 8	0.25 × 0.13 × 0.03 mm
*F*(000) = 1360	

### Data collection

**Table:**

Bruker APEXII CCD diffractometer	2515 reflections with *I* > 2σ(*I*)
φ and ω scans	*R*_int_ = 0.065
Absorption correction: multi-scan (*SADABS*; Bruker, 2009)	θ_max_ = 26.0°, θ_min_ = 2.0°
*T*_min_ = 0.979, *T*_max_ = 0.997	*h* = −12→12
52074 measured reflections	*k* = −9→9
3238 independent reflections	*l* = −50→50

### Refinement

**Table:**

Refinement on *F*^2^	0 restraints
Least-squares matrix: full	Hydrogen site location: inferred from neighbouring sites
*R*[*F*^2^ > 2σ(*F*^2^)] = 0.056	H-atom parameters constrained
*wR*(*F*^2^) = 0.148	*w* = 1/[σ^2^(*F*_o_^2^) + (0.0576*P*)^2^ + 2.9207*P*] where *P* = (*F*_o_^2^ + 2*F*_c_^2^)/3
*S* = 1.04	(Δ/σ)_max_ = 0.001
3238 reflections	Δρ_max_ = 0.66 e Å^−^^3^
218 parameters	Δρ_min_ = −0.25 e Å^−^^3^

### Special details

**Table:**

Geometry. All e.s.d.'s (except the e.s.d. in the dihedral angle between two l.s. planes) are estimated using the full covariance matrix. The cell e.s.d.'s are taken into account individually in the estimation of e.s.d.'s in distances, angles and torsion angles; correlations between e.s.d.'s in cell parameters are only used when they are defined by crystal symmetry. An approximate (isotropic) treatment of cell e.s.d.'s is used for estimating e.s.d.'s involving l.s. planes.

### Fractional atomic coordinates and isotropic or equivalent isotropic displacement parameters (Å^2^)

**Table:**

	*x*	*y*	*z*	*U*_iso_*/*U*_eq_	
O1	0.93462 (16)	−0.1993 (2)	0.07340 (4)	0.0440 (4)	
O2	0.97257 (17)	0.12811 (19)	0.10028 (4)	0.0407 (4)	
O3	0.84983 (16)	0.6264 (2)	0.18833 (4)	0.0470 (4)	
N1	0.9994 (2)	−0.2077 (3)	0.01922 (5)	0.0430 (5)	
C1	0.8083 (2)	−0.3616 (3)	0.03714 (6)	0.0427 (6)	
H1	0.7514	−0.3931	0.0546	0.051*	
C2	0.7879 (2)	−0.4169 (3)	0.00560 (6)	0.0454 (6)	
H2	0.7164	−0.4901	0.0008	0.054*	
C3	0.8727 (2)	−0.3645 (3)	−0.01884 (6)	0.0446 (6)	
H3	0.8595	−0.3993	−0.0408	0.054*	
C4	0.9750 (2)	−0.2626 (3)	−0.01099 (6)	0.0443 (6)	
H4	1.0329	−0.2280	−0.0280	0.053*	
C5	0.9177 (2)	−0.2561 (3)	0.04197 (5)	0.0357 (5)	
C6	1.0603 (2)	−0.1277 (3)	0.08188 (5)	0.0385 (5)	
H6	1.0927	−0.0579	0.0633	0.046*	
C7	1.1605 (3)	−0.2635 (3)	0.08878 (6)	0.0516 (7)	
H7A	1.1273	−0.3353	0.1063	0.077*	
H7B	1.2449	−0.2131	0.0955	0.077*	
H7C	1.1743	−0.3300	0.0690	0.077*	
C8	1.0369 (3)	−0.0192 (3)	0.11085 (6)	0.0413 (6)	
H8A	0.9807	−0.0781	0.1270	0.050*	
H8B	1.1225	0.0088	0.1214	0.050*	
C9	0.9480 (2)	0.2473 (3)	0.12354 (5)	0.0328 (5)	
C10	0.8926 (3)	0.3944 (3)	0.11212 (6)	0.0418 (6)	
H10	0.8755	0.4079	0.0894	0.050*	
C11	0.8624 (2)	0.5218 (3)	0.13372 (6)	0.0419 (6)	
H11	0.8244	0.6227	0.1260	0.050*	
C12	0.8880 (2)	0.5010 (3)	0.16646 (6)	0.0381 (5)	
C13	0.9423 (2)	0.3563 (3)	0.17803 (6)	0.0405 (6)	
H13	0.9588	0.3437	0.2008	0.049*	
C14	0.9732 (2)	0.2277 (3)	0.15657 (5)	0.0389 (5)	
H14	1.0114	0.1273	0.1645	0.047*	
C15	0.9434 (2)	0.7403 (3)	0.19842 (5)	0.0335 (5)	
C16	1.0758 (2)	0.7374 (3)	0.18908 (5)	0.0376 (5)	
H16	1.1078	0.6548	0.1744	0.045*	
C17	1.1612 (3)	0.8584 (3)	0.20167 (6)	0.0441 (6)	
H17	1.2521	0.8581	0.1955	0.053*	
C18	1.1157 (3)	0.9779 (3)	0.22293 (6)	0.0465 (6)	
H18	1.1751	1.0585	0.2316	0.056*	
C19	0.9830 (3)	0.9803 (3)	0.23162 (5)	0.0434 (6)	
H19	0.9510	1.0640	0.2460	0.052*	
C20	0.8970 (2)	0.8620 (3)	0.21951 (5)	0.0387 (5)	
H20	0.8060	0.8639	0.2256	0.046*	

### Atomic displacement parameters (Å^2^)

**Table:**

	*U*^11^	*U*^22^	*U*^33^	*U*^12^	*U*^13^	*U*^23^
O1	0.0434 (10)	0.0497 (10)	0.0389 (9)	−0.0070 (8)	0.0040 (7)	−0.0049 (7)
O2	0.0592 (10)	0.0341 (8)	0.0290 (8)	0.0042 (8)	−0.0059 (7)	0.0004 (6)
O3	0.0366 (9)	0.0428 (10)	0.0618 (11)	−0.0025 (8)	0.0069 (8)	−0.0159 (8)
N1	0.0424 (11)	0.0441 (12)	0.0424 (11)	0.0004 (9)	0.0015 (9)	−0.0029 (9)
C1	0.0396 (13)	0.0426 (14)	0.0459 (14)	0.0053 (11)	0.0021 (11)	0.0098 (11)
C2	0.0387 (13)	0.0353 (13)	0.0622 (16)	−0.0019 (11)	−0.0161 (12)	0.0006 (12)
C3	0.0472 (14)	0.0498 (14)	0.0368 (13)	0.0126 (12)	−0.0118 (11)	−0.0084 (11)
C4	0.0430 (14)	0.0494 (15)	0.0404 (13)	0.0033 (12)	0.0011 (11)	0.0014 (11)
C5	0.0401 (12)	0.0340 (12)	0.0330 (11)	0.0096 (10)	−0.0075 (10)	−0.0037 (9)
C6	0.0378 (13)	0.0424 (13)	0.0353 (12)	−0.0025 (11)	−0.0014 (10)	−0.0053 (10)
C7	0.0529 (16)	0.0536 (16)	0.0484 (14)	0.0071 (13)	−0.0012 (12)	−0.0074 (12)
C8	0.0508 (14)	0.0360 (13)	0.0372 (12)	0.0029 (11)	−0.0045 (10)	−0.0006 (10)
C9	0.0363 (12)	0.0306 (11)	0.0314 (11)	−0.0030 (9)	−0.0030 (9)	−0.0007 (9)
C10	0.0506 (14)	0.0385 (13)	0.0362 (12)	−0.0009 (11)	−0.0043 (11)	0.0079 (10)
C11	0.0430 (14)	0.0335 (12)	0.0493 (14)	0.0031 (11)	−0.0012 (11)	0.0057 (11)
C12	0.0315 (11)	0.0365 (12)	0.0461 (13)	−0.0038 (10)	0.0016 (10)	−0.0065 (10)
C13	0.0429 (13)	0.0452 (14)	0.0334 (12)	0.0002 (11)	−0.0040 (10)	−0.0040 (10)
C14	0.0470 (14)	0.0355 (12)	0.0343 (12)	0.0029 (11)	−0.0064 (10)	0.0017 (10)
C15	0.0376 (12)	0.0306 (11)	0.0322 (11)	−0.0006 (10)	−0.0030 (9)	0.0020 (9)
C16	0.0398 (13)	0.0362 (12)	0.0368 (12)	0.0040 (10)	0.0008 (10)	0.0029 (10)
C17	0.0384 (13)	0.0463 (14)	0.0477 (14)	−0.0033 (11)	−0.0069 (11)	0.0093 (12)
C18	0.0561 (16)	0.0392 (13)	0.0440 (13)	−0.0040 (12)	−0.0170 (12)	0.0032 (11)
C19	0.0605 (16)	0.0382 (13)	0.0314 (12)	0.0032 (12)	−0.0073 (11)	−0.0018 (10)
C20	0.0436 (13)	0.0390 (12)	0.0335 (12)	0.0068 (11)	−0.0007 (10)	0.0010 (10)

### Geometric parameters (Å, º)

**Table:**

O1—C5	1.375 (3)	C8—H8B	0.9900
O1—C6	1.432 (3)	C9—C14	1.384 (3)
O2—C9	1.372 (3)	C9—C10	1.387 (3)
O2—C8	1.416 (3)	C10—C11	1.386 (3)
O3—C15	1.376 (3)	C10—H10	0.9500
O3—C12	1.401 (3)	C11—C12	1.374 (3)
N1—C5	1.302 (3)	C11—H11	0.9500
N1—C4	1.335 (3)	C12—C13	1.368 (3)
C1—C2	1.380 (3)	C13—C14	1.391 (3)
C1—C5	1.404 (3)	C13—H13	0.9500
C1—H1	0.9500	C14—H14	0.9500
C2—C3	1.380 (4)	C15—C20	1.385 (3)
C2—H2	0.9500	C15—C16	1.387 (3)
C3—C4	1.354 (4)	C16—C17	1.395 (3)
C3—H3	0.9500	C16—H16	0.9500
C4—H4	0.9500	C17—C18	1.373 (4)
C6—C8	1.490 (3)	C17—H17	0.9500
C6—C7	1.511 (3)	C18—C19	1.382 (4)
C6—H6	1.0000	C18—H18	0.9500
C7—H7A	0.9800	C19—C20	1.377 (3)
C7—H7B	0.9800	C19—H19	0.9500
C7—H7C	0.9800	C20—H20	0.9500
C8—H8A	0.9900		
			
C5—O1—C6	117.96 (18)	O2—C9—C14	124.4 (2)
C9—O2—C8	116.94 (16)	O2—C9—C10	115.65 (19)
C15—O3—C12	118.77 (17)	C14—C9—C10	120.0 (2)
C5—N1—C4	116.5 (2)	C11—C10—C9	120.1 (2)
C2—C1—C5	116.3 (2)	C11—C10—H10	119.9
C2—C1—H1	121.9	C9—C10—H10	119.9
C5—C1—H1	121.9	C12—C11—C10	119.4 (2)
C1—C2—C3	119.1 (2)	C12—C11—H11	120.3
C1—C2—H2	120.4	C10—C11—H11	120.3
C3—C2—H2	120.4	C13—C12—C11	121.0 (2)
C4—C3—C2	118.9 (2)	C13—C12—O3	119.9 (2)
C4—C3—H3	120.6	C11—C12—O3	118.9 (2)
C2—C3—H3	120.6	C12—C13—C14	120.1 (2)
N1—C4—C3	124.0 (2)	C12—C13—H13	119.9
N1—C4—H4	118.0	C14—C13—H13	119.9
C3—C4—H4	118.0	C9—C14—C13	119.4 (2)
N1—C5—O1	119.5 (2)	C9—C14—H14	120.3
N1—C5—C1	125.1 (2)	C13—C14—H14	120.3
O1—C5—C1	115.4 (2)	O3—C15—C20	115.1 (2)
O1—C6—C8	106.72 (19)	O3—C15—C16	124.4 (2)
O1—C6—C7	110.2 (2)	C20—C15—C16	120.5 (2)
C8—C6—C7	112.2 (2)	C15—C16—C17	118.6 (2)
O1—C6—H6	109.2	C15—C16—H16	120.7
C8—C6—H6	109.2	C17—C16—H16	120.7
C7—C6—H6	109.2	C18—C17—C16	121.0 (2)
C6—C7—H7A	109.5	C18—C17—H17	119.5
C6—C7—H7B	109.5	C16—C17—H17	119.5
H7A—C7—H7B	109.5	C17—C18—C19	119.6 (2)
C6—C7—H7C	109.5	C17—C18—H18	120.2
H7A—C7—H7C	109.5	C19—C18—H18	120.2
H7B—C7—H7C	109.5	C20—C19—C18	120.4 (2)
O2—C8—C6	108.52 (18)	C20—C19—H19	119.8
O2—C8—H8A	110.0	C18—C19—H19	119.8
C6—C8—H8A	110.0	C19—C20—C15	119.9 (2)
O2—C8—H8B	110.0	C19—C20—H20	120.1
C6—C8—H8B	110.0	C15—C20—H20	120.1
H8A—C8—H8B	108.4		
			
C5—C1—C2—C3	−1.1 (3)	C10—C11—C12—C13	−0.3 (4)
C1—C2—C3—C4	1.2 (4)	C10—C11—C12—O3	−176.2 (2)
C5—N1—C4—C3	−0.5 (4)	C15—O3—C12—C13	86.0 (3)
C2—C3—C4—N1	−0.4 (4)	C15—O3—C12—C11	−98.0 (3)
C4—N1—C5—O1	−177.7 (2)	C11—C12—C13—C14	0.4 (4)
C4—N1—C5—C1	0.7 (3)	O3—C12—C13—C14	176.3 (2)
C6—O1—C5—N1	−17.0 (3)	O2—C9—C14—C13	−179.0 (2)
C6—O1—C5—C1	164.4 (2)	C10—C9—C14—C13	0.2 (3)
C2—C1—C5—N1	0.1 (3)	C12—C13—C14—C9	−0.4 (4)
C2—C1—C5—O1	178.6 (2)	C12—O3—C15—C20	178.8 (2)
C5—O1—C6—C8	157.85 (19)	C12—O3—C15—C16	−1.8 (3)
C5—O1—C6—C7	−80.0 (2)	O3—C15—C16—C17	−178.6 (2)
C9—O2—C8—C6	−177.21 (19)	C20—C15—C16—C17	0.7 (3)
O1—C6—C8—O2	−75.0 (2)	C15—C16—C17—C18	0.1 (3)
C7—C6—C8—O2	164.1 (2)	C16—C17—C18—C19	−1.0 (4)
C8—O2—C9—C14	−4.7 (3)	C17—C18—C19—C20	1.1 (3)
C8—O2—C9—C10	176.0 (2)	C18—C19—C20—C15	−0.2 (3)
O2—C9—C10—C11	179.2 (2)	O3—C15—C20—C19	178.7 (2)
C14—C9—C10—C11	−0.1 (4)	C16—C15—C20—C19	−0.7 (3)
C9—C10—C11—C12	0.1 (4)		

### Hydrogen-bond geometry (Å, º)

Cg1 and Cg2 are the centroids of the N1/C4/C3/C2/C1/C5 and C15–C20 rings, respectively.

**Table:**

*D*—H···*A*	*D*—H	H···*A*	*D*···*A*	*D*—H···*A*
C2—H2···*Cg*1^i^	0.95	2.85	3.667 (3)	145
C14—H14···*Cg*2^ii^	0.95	2.86	3.733 (2)	152
C19—H19···*Cg*2^iii^	0.95	2.97	3.857 (2)	156

Symmetry codes: (i) −*x*+3/2, *y*−1/2, *z*; (ii) *x*, *y*−1, *z*; (iii) −*x*+2, *y*+1/2, −*z*+1/2.
